# Sepsis Outcome after Major Abdominal Surgery Does Not Seem to Be Improved by the Use of Pentameric Immunoglobulin IgM: A Single-Center Retrospective Analysis

**DOI:** 10.3390/jcm12216887

**Published:** 2023-10-31

**Authors:** Alessandro Perrella, Luca Rinaldi, Ilaria Guarino, Francesca Futura Bernardi, Maurizio Castriconi, Carmine Antropoli, Pia Clara Pafundi, Pierpaolo Di Micco, Marina Sarno, Nicolina Capoluongo, Giuseppina Minei, Marco Perrella, Antonio Frangiosa, Annalisa Capuano

**Affiliations:** 1 Department of Emerging Infectious Disease at High Countagiousness, AORN Ospedali dei Colli, P.O.D. Cotugno, 80131 Naples, Italy; marinasarno92@gmail.com (M.S.); nicolina.capoluongo@ospedalideicolli.it (N.C.); marco.perrella@aocardarelli.it (M.P.); 2 Department of Medicine and Health Sciences, “Vincenzo Tiberio” Università degli Studi del Molise, 86000 Campobasso, Italy; luca.rinaldi@unicampania.it (L.R.); piaclara.pafundi88@gmail.com (P.C.P.); 3 Intensive Care Unit, AORN A. Cardarelli, 80131 Naples, Italy; 4 Directorate-General for Health Protection, Campania Region, 80143 Naples, Italy; bernardi.francesca.futura@gmail.com; 5 Emergency Surgery, AORN A. Cardarelli, 80131 Naples, Italy; 6 Abdominal Surgery, AORN A. Cardarelli, 80131 Naples, Italy; 7 Department of Internal Medicine, Rizzoli Hospital, 80076 Naples, Italy; pdimicco@libero.it; 8 Post Operative Intensive Care Division, A. Cardarelli Hospital, 80131 Naples, Italy; antonio.frangiosa@aocardarelli.it; 9 Campania Regional Centre for Pharmacovigilance and Pharmacoepidemiology, 80138 Naples, Italy; annalisa.capuano@unicampania.it

**Keywords:** pentaglobin, sepsis, infection, abdominal infection

## Abstract

Background: Sepsis still represents a major public health issue worldwide, and the immune system plays a main role during infections; therefore, its activity is mandatory to resolve this clinical condition. In this report, we aimed to retrospectively verify in a real-life setting the possible usefulness of pentameric IgM plus antibiotics in recovering patients with sepsis after major abdominal surgery. Materials/methods: We reviewed, from January 2013 until December 2019, all adult patients admitted to the ICU for sepsis or septic shock (2) after major abdominal surgery. Among these patients, were identified those that, according to legal indication and licenses in Italy, were treated with pentameric IgM plus antibiotics (Group A) or with antibiotics alone (Group B). The following parameters were evaluated: blood gas analysis, lactate, CRP, procalcitonin, endotoxin activity, liver and renal function, coagulation and blood cell count at different time points (every 48 h for at least 7 days). Differences between groups were analyzed using Fisher’s exact test or a chi-square test for categorical variables. A Mann–Whitney U test or Kruskal–Wallis test were instead been performed to compare continuous variables. Univariate and multivariate analysis were also performed. Results: Over a period of 30 months, 24 patients were enrolled in Group A and 20 patients in Group B. In those subjects, no statistical differences were found in terms of bacterial or fungal infection isolates, when detected in a blood culture test, or according to inflammatory index, a score, lactate levels and mortality rate. A 48 h response was statistically more frequent in Group B than in Group A, while no differences were found in other clinical and laboratory evaluations. Conclusions: Based on our results, the use of pentameric IgM does not seem to give any clinical advantages in preventing sepsis after major abdominal surgery.

## 1. Introduction

Sepsis still represents a major public health concern worldwide, being characterized by organ dysfunction and related dysregulated host response [1,2,3]. The current approach to sepsis includes the early eradication of septic foci, administration of anti-infective agents and maintenance of hemodynamic stability using fluid administration and vasopressors [1,3,4]. This treatment is the cornerstone for sepsis and, in particular, septic shock prevention [4]. The main difficulties in treating sepsis are related to its complexity, which depends on the types of infectious microorganisms (such as bacteria and fungi), with related differences in terms of virulence and resistance to antibiotics. Further, the different infected body sites, especially in patients with several comorbidities, may have a significant impact on the outcome [1]. Coupled with this evidence, we should also consider that these patients may vary in their ability to respond to infection (due to hyperinflammation or immune paralysis) and treatments increasing the complexity of the disease. Regarding immune paralysis, sepsis has been defined as a life-threatening organ dysfunction caused by a dysregulated host response to infection [1]. The host response is characterized by inflammatory storm and concurrent immunosuppression, which promote tissue damage, the down-regulation of activating cell surface molecules, T cell depletion and increased apoptosis of immune cells [5]. This imbalance in the immune system may determine a profound dysfunction of the innate and adaptive immunity [6] and play a role in patient outcomes, particularly in the elderly and patients with pre-existing immune disorders. Since the use of only anti-inflammatory drugs has failed in reducing mortality, the use of therapies designed to re-establish the immune system seems plausible. Several therapeutic options have been suggested to improve the outcome of sepsis and its clinical complications as septic shock and multiple organ dysfunction syndrome; more recently, it has been proposed that improving the opsonizing ability of the immune system may reduce bacterial virulence. In this field, despite guidelines not recommending the use of immunomodulatory treatment based on pentameric IgM (Pentaglobin, an immunoglobulin M-enriched immunoglobulin), this treatment schedule has been proposed in some scientific reports [2,7]. The rationale for this kind of therapeutic approach is controversial because no clear consensus on its real benefits in clinical practice is provided. The advantage of this therapy relies on its pleiotropic effects on inflammation and the immune system [8,9]. Further, evidence from in vitro and in vivo studies and few clinical data have supported its use [7]. Previous guidelines suggested against the use of polyclonal intravenous immunoglobulins in sepsis, but based on weak efficacy data [2]. However, results from recent trials and systematic meta-analyses indicate that intravenous IgM-enriched immunoglobulins may be effective in sepsis [9,10,11]. The aim of our retrospective observational study was to verify, in a real-life setting, whether pentameric IgM-enriched immunoglobulin may improve clinical outcome and survival when early associated with antibiotic treatment in patients with sepsis admitted to an intensive care unit (ICU).

## 2. Methods

### 2.1. Study Design

A retrospective medical record review was performed to include anonymized data on patients admitted for sepsis or septic shock after major abdominal surgery to the ICU of our hospital (AORN Ospedali dei Colli, Napoli, Italia and AORN A Cardarelli, Napoli, Italy) for the period from January 2013 to December 2019. The data were related to adult patients (≥18 years) diagnosed and treated for sepsis or septic shock, according to international guidelines and or national/regional guidelines indicated throughout the retrospective period of evaluation [1,2,11,12]. Pentameric IgM, when used, was used according to its license of use in Italy (AIFA Italian Drug Regulatory Agency).

**Figure 1:**
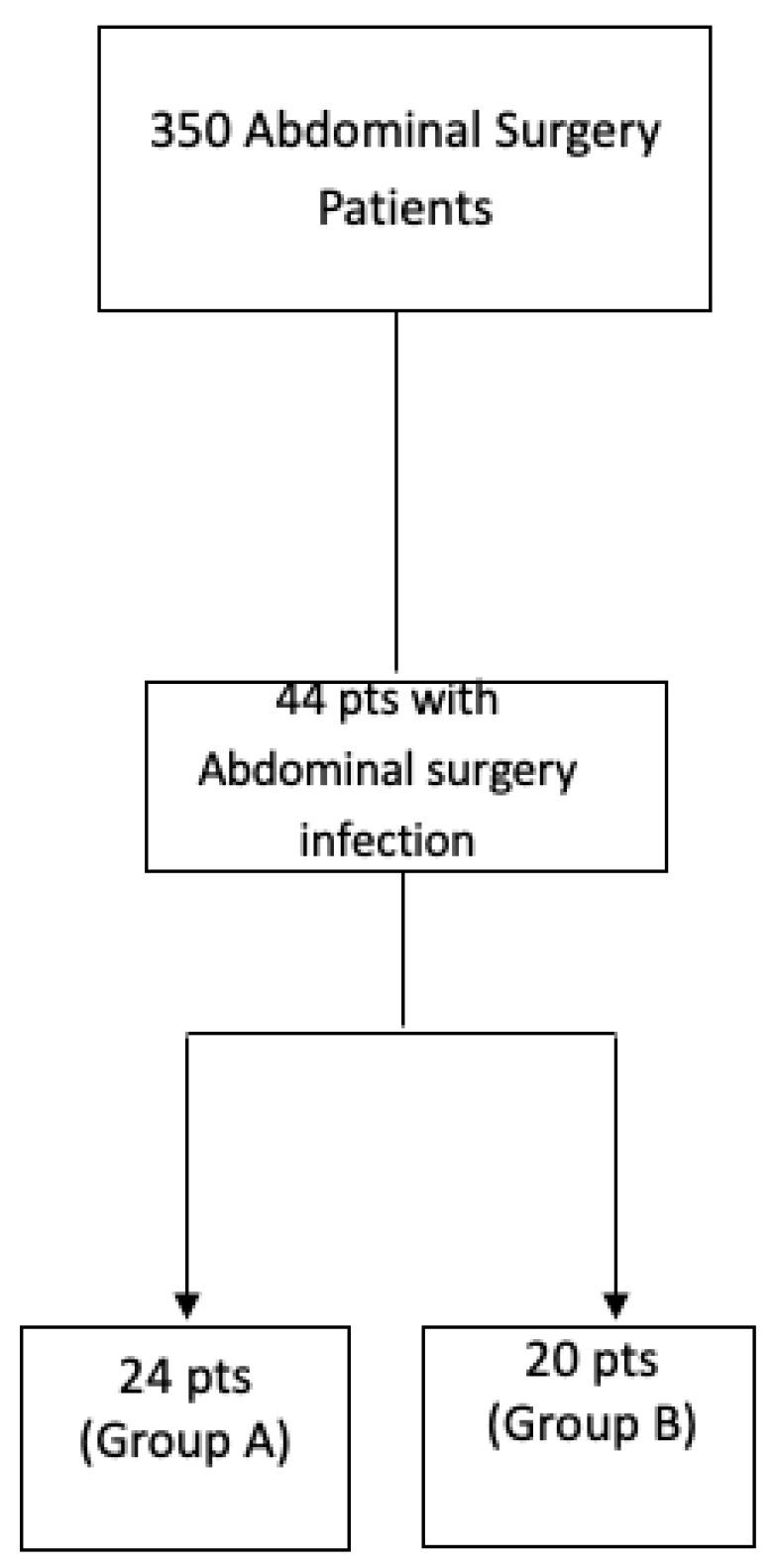
Flowchart of evaluated patients in the retrospective analysis (January 2013–March 2019).

Particularly, the collected data were divided into two groups: Group A patients underwent pentameric IgM plus antibiotics and patients selected from the same cohort of surgical procedures but treated with antibiotics alone comprised Group B (Figure 1). Demographic data and relevant comorbidities were recorded for all patients in terms of laboratory markers and clinical outcomes. According to clinical practice and local guidelines, the following parameters were retrospectively evaluated every 48 h: blood gas analysis, lactate, CRP, procalcitonin, endotoxin activity, liver and renal function, coagulation and blood cell count. Empirical antibiotic treatment was planned for all patients and was based on their legal indications and license of use in Europe and Italy, as well as regional and hospital guidelines, or after infectious disease consultant assessment. Mainly, all empiric treatments in patients with abdominal infections were based on the following schedules: tigecycline + piperacilin/tazobactam, or according to hospital or regional guidelines and their updates (https://www.regione.campania.it/assets/documents/linee-indirizzo-terapia-antibiotica.pdf accessed on 24 August 2023). This therapeutic schedule was modified according to scientific evidence from the infectious disease group in surgery at AORN A. Cardarelli [13] and national/regional guidelines, as well as microbiological isolates when required. Antifungals were used when required according to a previous score system [7] and were based on azoles or liposomal amphotericien B, according to risk factors. The laboratory parameters were evaluated at a centralized laboratory; particularly, CRP and procalcitonin were assessed according to previous evidence [7,13]. Endotoxin activity is measured as the relative oxidative burst of primed neutrophils as detected using chemiluminescence. The assay’s output is expressed on a scale from 0 (absent) to 1 (maximal) and categorized as “low” (<0.4 units), “intermediate” (0.4–0.59 units) or “high” (≥0.6 units). Endotoxin activity assay was performed within one hour of blood collection according to the vacutainer systems (Becton Dickinson, Franklin Lakes, NJ, USA).

### 2.2. Study Endpoints

The primary endpoint was to evaluate the 48 h clinical and laboratory response (reduction in fever, inotrope drugs use, CRP and leukocyte as well as increase in blood pressure) to antibiotics and pentameric immunoglobulin. The secondary endpoint was mortality rate and sepsis resolution (withdrawal of inotrope drugs, absence of fever, reduction in inflammatory parameters, discharge from ward within 28th day).

### 2.3. Ethics Approval

According to the local legislation, a retrospective study does not require ethical approval. For the use of retrospective data, this study was conducted in accordance with the Declaration of Helsinki 1975 and its later amendments. All patients’ data were fully anonymized and were analyzed retrospectively. For this type of study, formal consent was not required according to the current national guidelines established by the Italian Medicines Agency, and according to the Italian Data Protection Authority. Neither was ethical committee approval nor informed consent required for anonymized data, as confirmed and approved by the Ethical Committee of “Aziende Ospedaliere di Rilievo Nazionale e di Alta Specializzazione—A.Cardarelli/Santobono—Pausilipon” as part of a larger study (Protocol Number 00000926 of 11 January 2022).

### 2.4. Statistical Analysis 

Statistical analysis was performed looking for differences between groups by using the Fisher’s exact test or chi-square test for categorical variables. The Mann–Whitney U test or Kruskal–Wallis test was instead performed to compare continuous variables. Univariate and multivariate analysis were also performed. *p* values below 0.05 were considered statistically significant. All analyses were performed using the SPSS software (IBM, Armonk, NY, USA), version 24. Data are shown as either medians and ranges, in the case of continuous variables, or numbers and percentages, for categorical variables.

## 3. Results

Overall, data related to 44 patients (29 men and 15 women) were retrieved from medical records. Specifically, 24 patients (18 men and 6 women) were evaluated as eligible in Group A and 20 patients (11 men and 9 women) in Group B. The clinical data of all patients are summarized in Table 1. No difference was observed for demographic (age and sex) and clinical characteristics (BMI, smoke, potus, diabetes and metabolic syndrome) at baseline between groups (Table 2). No statistical difference was found in terms of bacterial or fungal infections, when detected in a blood culture test, or in inflammatory index, SOFA score, lactate levels and mortality rate (Table 2). The primary endpoint showed that a 48 h response was statistically more frequent in Group B than in Group A, while no difference in the secondary endpoint was found. The only further statistically difference was in the median ICU stay, which was prolonged (over 14 days) in patients of Group A compared to those in Group B (Table 2).

## 4. Discussion

Sepsis and its complications as septic shock remain a critical issue in healthcare because they are still associated with increased morbidity and the mortality of affected inpatients in emergency departments and in ICUs. Nevertheless, a multidisciplinary clinical approach and tailored treatment based on antibiotics, antifungals when necessary, fluids, steroids and vasopressors represents a gold standard that may improve clinical outcomes and reduce mortality [2]. The use of immunoglobulins to treat sepsis is mainly based on the rationale of modulating the inflammatory reaction and supporting the immune system in the fight against pathogens [7]. Preclinical evidence showed that the infusion of IgM-enriched immunoglobulins can shift the inflammatory response toward an anti-inflammatory profile [7]. IgM-enriched immunoglobulins can normalize capillary perfusion by reducing the leukocyte adhesion in experimental models [14]. Moreover, IgM-enriched immunoglobulins were shown to enhance the anti-inflammatory response by increasing IL-10 levels and reducing the TNF-alpha in the bronchoalveolar lavage fluid of pneumonia models [15,16,17,18]. In a clinical setting, the use of intravenous human immunoglobulin (IVIG) to improve the sepsis outcome of patients who had undergone abdominal surgery is still matter of discussion [7,19]. In our study, according to other literature’s evidence too [7,11], we found that after major abdominal surgery, a therapeutic approach based on pentameric IgM plus antibiotic treatment did not seem to improve the natural history of sepsis, in terms of outcome and mortality compared to a therapy schedule based on the sole antibiotics. On the contrary, a meta-analysis, including 15 randomized clinical trials (712 patients) and 4 cohort studies (818 patients), found a reduction in mortality rates with IgM-enriched immunoglobulin for sepsis (Risk Ratio 0.60; 95%CI 0.52–0.69). Subgroup analyses also showed that these findings were consistent in reference to treatment duration, daily dose, total dose, variety of disease, severity scores, follow-up duration, study design and year of publication [20]. However, this meta-analysis mainly compared IgM-enriched immunoglobulin with a placebo. Further, we also did not find any differences in terms of the endotoxin activity assay between the two groups. Indeed, endotoxin is an expression of systemic inflammation due to abdominal infection being sustained via Gram-negative bacteria [21]. Despite the mechanisms related to the possible effects of pentameric IgM on different settings of patients still not being clear, our findings, even if based on a retrospective study, underline the importance of a better and more exhaustive evaluation of the use of pentameric IgM, instead of too-easy empiric use.

Our study also carries some limitations. First, the small sample size may under-power the detection of differences along some parameters, such as mortality or other major outcomes. Further, it is a single-center retrospective study of a tertiary care hospital with well-known expertise in surgical procedures of high complexity. Therefore, our results should be considered as exploratory and as the first step into deeper knowledge on how and when to use pentameric immunoglobulin.

## 5. Conclusions

In conclusion, based on our results, we can deduce that the use of pentameric IgM for sepsis, retrospectively evaluated after major abdominal surgery, did not seem to give any clinical advantage in the short term and by 28 days when death and duration of stay in an ICU are considered as outcomes. Given the small sample size, these results must be seen as exploratory and need to be confirmed by other larger-population-based studies.

## Figures and Tables

**Table 1:** Baseline characteristics of the entire cohort of study (*n* = 44).

Parameter	
Age (yrs), median [IQR]	53.5 [45.2–59.7]
Sex, *n* (%)	
Male	29 (65.9)
Female	15 (34.1)
BMI, median [IQR]	23.5 [22–25]
Smoke, *n* (%)	22 (50)
Potus, *n* (%)	12 (27.3)
Diabetes, *n* (%)	8 (18.2)
Metabolic syndrome, *n* (%)	7 (15.9)
Lactates at EAB, median [IQR]	2.15 [1.73–3]
SOFA score, median [IQR]	8 [7–8]
Leukocytosis, median [IQR]	
Baseline	20.5 [18–22.3]
72 h	18 [16–20]
96 h	16 [13.2–18]
PCT, median [IQR]	
Baseline	4.5 [2–8]
48 h	5 [3–6]
EAA, median [IQR]	
Baseline	0.6 [0.5–0.7]
72 h	0.51 [0.5–0.6]
PCR, median [IQR]	
Baseline	183.5 [147–254]
48 h	124 [109–181]
Therapy, *n* (%)	
Pentaglobin + antibiotic	24 (54.5)
Only antibiotic	20 (45.5)
Major abdominal surgery, *n* (%)	34 (77.3)
Inotropes, *n* (%)	17 (38.6)
Inotrope drugs, *n* (%)	21 (47.7)
Acidosis at EGA, *n* (%)	23 (52.3)
ICU stay over 15 days, *n* (%)	21 (47.7)
Response at 48 h, *n* (%)	23 (52.3)
Exitus, *n* (%)	11 (25)
Infected devices, *n* (%)	15 (34.1)
Fungal infections, *n* (%)	4 (9.1)
Bacteria, *n* (%)	
Gram-negative	35 (79.5)
Gram-positive	9 (20.5)

In the table are reported all analyzed parameters during ICU stay. Data are expressed as either numbers and percentages or medians and interquartile ranges (IQR). EAA means endotoxin activity assay. Potus means alcohol abuse. Gram-negative were as follows: (*Klebsiella* sp 12 pts, *Klebsiella* KPC 5 pts, *E. coli* 3 pts, *Enterobacter aer*. 4 pts, *Pseudomonas aer*. 4 pts, *Acinetobacter baumannii* MDR 7 pts), Gram-positive were *Enterococcus faecalis* 4 pts, *Enterococcus faecium* 5 pts (all isolated Enterococci were not vancomycin-resistant).

**Table 2:** Baseline characteristics according to pentaglobin therapy: univariate and multivariate analysis (*n* = 44).

	Univariate Analysis		
Parameter	Pentaglobin + Antibiotic (n = 24)	Antibiotic (n = 20)	*p*
Age (yrs), median [IQR]	48.5 [45.2–57]	57.5 [47–61.7]	0.125
Sex, n (%)			0.210
Male	18 (75)	11 (65)	
Female	6 (25)	9 (35)	
BMI, median [IQR]	23 [22–25]	24 [23–26]	0.204
Smoke, n (%)	14 (58.3)	8 (40)	0.364
Potus, n (%)	5 (20.8)	7 (35)	0.329
Diabetes, n (%)	3 (12.5)	5 (25)	0.436
Metabolic syndrome, n (%)	2 (8.3)	5 (25)	0.217
SOFA score, median [IQR]	8 [7–9]	8 [7–8]	0.360
Lactates at EAB, median [IQR]	2.3 [1.85–3]	2 [1.55–3.75]	0.849
Leukocytosis, median [IQR]			
Baseline	19.5 [17.2–22.3]	21 [19–22.8]	0.293
72 h	18 [16–20.7]	18 [16–20]	0.785
96 h	15.3 [12.2–18]	16 [15.2–18]	0.414
PCT, median [IQR]			
Baseline	3.5 [2–7.5]	5 [3.13–8.75]	0.129
48 h	5 [2–7]	5 [4–6]	0.403
EAA, median [IQR]			
Baseline	0.6 [0.5–0.7]	0.55 [0.4–0.7]	0.457
72 h	0.56 [0.5–0.6]	0.5 [0.5–0.67]	0.772
PCR, median [IQR]			
Baseline	202 [153–242.2]	163.5 [135.2–258.2]	0.548
48 h	123 [108.2–174.7]	146 [110–191.2]	0.333
Major abdominal surgery, n (%)	24 (70.8)	20 (85)	0.402
Inotrope, n (%)	11 (45.8)	6 (30)	0.359
Inotrope drugs, n (%)	14 (58.3)	7 (35)	0.143
Acidosis at EGA, n (%)	12 (50)	11 (55)	0.771
ICU stay over 14 days, n (%)	17 (70.8)	4 (20)	0.001
Response at 48 h, n (%)	6 (25)	17 (85)	0.000
Exitus, n (%)	6 (25)	5 (25)	1.000
Infected devices, n (%)	11 (45.8)	4 (20)	0.111
Fungal infections, n (%)	2 (8.3)	2 (10)	1.000
Bacteria, n (%)			
Gram-negative	20 (83.3)	15 (75)	0.710
MDR ^	4 (16.6)	3(15)	n.s.
Gram-positive	4 (16.7)	5 (25)	0.710

Table shows the results related to the analyzed variables in both groups. Statistically significant differences were found in ICU stay over 14 days and response at 48 h. No differences have been found in bacterial infections. ^ Gram-negative MDR were *Acinetobacter baumannii* MDR in 7 cases. In this case, the following therapeutic schedule was used: colimycine plus tigecycline plus meropenem). Fungal infections were due to Candida albicans species being resistant to azoles. Data are expressed as either numbers and percentages or medians and interquartile ranges (IQR). EAA means endotoxin activity assay. Inotrope drugs refers to the combination of vasoactive drugs as noradrenalin and dobutamine used according to references [13,14] (norepinephrine >0.05 μg/kg/minute; dopamine > 10 μg/kg/minute). Infected devices refers to central vein catheters. Potus means alcohol abuse. Major abdominal surgery included hepatectomy 8 pts, cephalic duodenopancreatectomy 9 pts, segmental resection of the duodenum 2 pts, total gastrectomy 5 pts, abdominal abscess 20 pts. n.s.—not significant.

## Data Availability

Data not available outside of the Hospital.

## References

[1] Singer M., Deutschman C.S., Seymour C.W., Shankar-Hari M., Annane D., Bauer M., Bellomo R., Bernard G.R., Chiche J.-D., Coopersmith C.M. (2016). The Third International Consensus Definitions for Sepsis and Septic Shock (Sepsis-3). JAMA.

[2] Rhodes A., Evans L.E., Alhazzani W., Levy M.M., Antonelli M., Ferrer R., Kumar A., Sevransky J.E., Sprung C.L., Nunnally M.E. (2017). Surviving Sepsis Campaign: International Guidelines for Management of Sepsis and Septic Shock:2016. Crit. Care Med..

[3] Kyriazopoulou E., Leventogiannis K., Norrby-Teglund A., Dimopoulos G., Pantazi A., Orfanos S.E., Rovina N., Tsangaris I., Gkavogianni T., Botsa E. (2017). Macrophage activation-like syndrome: An immunological entity associated with rapid progression to death in sepsis. BMC Med..

[4] Artenstein A.W., Higgins T.L., Opal S.M. (2013). Sepsis and scientific revolutions. Crit. Care Med..

[5] Tang B.M., Huang S.J., McLean A.S. (2010). Genome-wide transcription profiling of human sepsis: A systematic review. Crit. Care.

[6] Tamayo E., Fernandez A., Almansa R., Carrasco E., Heredia M., Lajo C., Goncalves L., Gomez-Herreras J.I., de Lejarazu R.O., Bermejo-Martin J.F. (2011). Pro- and anti-inflammatory responses are regulated simultaneously from the first moments of septic shock. Eur. Cytokine Netw..

[7] Alejandria M.M., Lansang M.A.D., Dans L.F., Mantaring J.B. (2013). Intravenous immunoglobulin for treating sepsis, severe sepsis and septic shock. Cochrane Database Syst. Rev..

[8] Werdan K., Pilz G., Bujdoso O., Fraunberger P., Neeser G., Schmieder R.E., Viell B., Marget W., Seewald M., Walger P. (2007). Score-based immunoglobulin G therapy of patients with sepsis: The SBITS study. Crit. Care Med..

[9] Cavazzuti I., Serafni G., Busani S., Rinaldi L., Biagioni E., Buoncristiano M., Girardis M. (2014). Early therapy with IgM-enriched polyclonal immunoglobulin in patients with septic shock. Intensive Care Med..

[10] Giamarellos-Bourboulis E.J., Tziolos N., Routsi C., Katsenos C., Tsangaris I., Pneumatikos I., Vlachogiannis G., Theodorou V., Prekates A., Antypa E. (2016). Improving outcomes of severe infections by multidrug-resistant pathogens with polyclonal IgM-enriched immunoglobulins. Clin. Microbiol. Infect..

[11] Dellinger R.P., Rhodes A., Evans L., Alhazzani W., Beale R., Jaeschke R., Machado F.R., Masur H., Osborn T., Parker M.M. (2013). Surviving Sepsis Campaign Guidelines Committee including the Pediatric Subgroup. Surviving Sepsis Campaign: International guidelines for management of severe sepsis and septic shock: 2012. Crit. Care Med..

[12] https://www.regione.campania.it/assets/documents/linee-indirizzo-terapia-antibiotica.pdf.

[13] Perrella A., Esposito C., Amato G., Perrella O., Migliaccio C., Pisaniello D., Calise F., Cuomo O., Santaniello W. (2016). Antifungal prophylaxis with liposomal amphotericin B and caspofungin in high-risk patients after liver transplantation: Impact on fungal infections and immune system. Infect. Dis..

[14] Prescott H.C., Sussman J.B., Wiersinga W.J. (2020). Postcritical illness vulnerability. Curr. Opin. Crit. Care.

[15] Kakoullis L., Pantzaris N.D., Platanaki C., Lagadinou M., Papachristodoulou E., Velissaris D. (2018). The use of IgM-enriched immunoglobulin in adult patients with sepsis. J. Crit. Care.

[16] Barratt-Due A., Sokolov A., Gustavsen A., Hellerud B.C., Egge K., Pischke S.E., Lindstad J.K., Pharo A., Castellheim A., Thorgersen E.B. (2013). Polyvalent immunoglobulin signifcantly attenuated the formation of IL-1β in Escherichia coli-induced sepsis in pigs. Immunobiology.

[17] Hofman J.N., Fertmann J.M., Vollmar B., Laschke M.W., Jauch K.W., Menger M.D. (2008). Immunoglobulin M-enriched human intravenous immunoglobulins reduce leukocyte-endothelial cell interactions and attenuate microvascular perfusion failure in normotensive endotoxemia. Shock.

[18] Vaschetto R., Clemente N., Pagni A., Esposito T., Longhini F., Mercalli F., Boggio E., Boldorini R., Chicchetti A., Dianzani U. (2017). A double blind randomized experimental study on the use of IgM-enriched polyclonal immunoglobulins in an animal model of pneumonia developing shock. Immunobiology.

[19] Rodríguez A., Rello J., Neira J., Maskin B., Ceraso D., Vasta L., Palizas F. (2005). Effects of high-dose of intravenous immunoglobulin and antibiotics on survival for severe sepsis undergoin surgery. Shock.

[20] Cui J., Wei X., Lv H., Li Y., Li P., Chen Z., Liu G. (2019). The clinical efficacy of intravenous IgM-enriched immunoglobulin (pentaglobin) in sepsis or septic shock: A meta-analysis with trial sequential analysis. Ann. Intensive Care.

[21] Perrella A., Carannante N., Capoluongo N., Mascolo A., Capuano A. (2023). Endotoxin: Structure Source and Effects. Endotoxin Induced-Shock: A Multidisciplinary Approach in Critical Care.

